# Mid-Term Outcomes of Laparoscopic Gastric Greater Curvature Plication versus Roux-en-Y Gastric Bypass: Weight Loss, Gastrointestinal Symptoms, and Health-Related Quality of Life

**DOI:** 10.3390/medicina58010064

**Published:** 2021-12-31

**Authors:** Rita Gudaityte, Agne Kavaliauskaite, Almantas Maleckas

**Affiliations:** 1 Department of Surgery, Medical Academy, Lithuanian University of Health Sciences, 44307 Kaunas, Lithuania; agnekavaliauskait@gmail.com (A.K.); Almantas.Maleckas@lsmu.lt (A.M.); 2 Department of Surgery, Institute of Clinical Sciences, Sahlgrenska Academy, University of Gothenburg, 405 30 Gothenburg, Sweden

**Keywords:** bariatric surgery, laparoscopic gastric greater curvature plication, Roux-en-Y gastric bypass, weight loss, gastrointestinal symptoms, quality of life

## Abstract

*Background and Objectives*: Laparoscopic gastric greater curvature plication (LGGCP) is considered to be less invasive, technically simpler, and less costly. Few studies have compared LGGCP to gastric bypass. The aim of this prospective study was to evaluate the mid-term outcomes of LGGCP such as weight loss, gastrointestinal symptoms, and health-related quality of life (HRQoL) in comparison to laparoscopic Roux-en-Y gastric bypass (LRYGB). *Materials and Methods*: Between 2017 April and 2018 December, 112 patients were included in the study. Fifty patients had LGGCP, and sixty-two patients underwent LRYGB. Demographics, comorbidities, complications, percentage of excess body mass index loss (%EBMIL), gastrointestinal symptoms (GSRS questionnaire), and HRQoL (EQ-5D-3L questionnaire) were analysed. Gastrointestinal symptoms and HRQoL data are presented as the mean and median with the interquartile range (25th–75th percentile). Follow-up at 1 year and 3 year was performed. *Results*: The follow-up rate was 96.4% and 92.9%, 1 year and 3 year after surgery, respectively. Mean (SD) %EBMIL 1 year after surgery was 59.05 (25.34) in the LGGCP group and 82.40 (19.03) in the LRYGB group (*p* < 0.001) and 3 year after was 41.44 (26.74) and 75.59 (19.14), respectively (*p* < 0.001). The scores of all gastrointestinal symptoms measured by the GSRS questionnaire significantly decreased 3 year after both procedures, except reflux after LGGCP. Patients 3 year after LGGCP had a significantly lower abdominal pain score as compared to patients after LRYGB (1.01; 1.0 (1.0–1.0) and 1.20; 1.0 (1.0–1.33), respectively (*p* < 0.001); however, LGGCP resulted in significantly more GERD symptoms (1.79; 1.25 (1.0–2.5) and 1.18; 1.0 (1.0–1.0), respectively (*p* < 0.001)). Three years after surgery, the quality of life was significantly lower in the LGGCP group (0.762; 0.779 (0.690–0.794) and 0.898; 1.000 (0.783–1.000), respectively (*p* < 0.001)). *Conclusions*: Three years after surgery, LGGCP patients lost significantly less weight, had less abdominal pain and more reflux symptoms, and a lower quality of life as compared to LRYGB patients.

## 1. Introduction

The prevalence of obesity and associated diseases has dramatically increased during the last few decades [1]. Currently, bariatric surgery is the most effective method to treat morbid obesity. It results in good long-term weight loss and acceptable control of comorbidities and improves the quality of life [2]. Even though bariatric surgery has been performed for more than 50 year, still new procedures are evolving. There is a need for less invasive, but efficient methods. Laparoscopic gastric greater curvature plication (LGGCP) may fulfil some of these requirements because there is no tissue transection or removal. It was reintroduced more than 10 year ago by Talebpour and Amoli [3] and gained some popularity because of the technical simplicity and low cost. 

LGGCP was considered to be an improvement of laparoscopic sleeve gastrectomy (LSG) as there was no need to resect the stomach. Thus, most of the comparative studies that were performed during last 10 year included LGGCP and LSG [4]. A recent systematic review concluded that LGGCP as compared to LSG had a higher risk of complications and reoperations and resulted in lower %EWL, while the difference in cost was only modestly in favour of LGGCP [5]. However, few studies have compared LGGCP to laparoscopic gastric bypass [6,7]. 

Most of the studies analysed complications, weight loss, reoperations, remission of comorbidities, and reflux between LGGCP and other procedures. However, no data are available about the impact of LGGCP on the quality of life and gastrointestinal symptoms other than reflux. The aim of this prospective study was to evaluate mid-term outcomes of LGGCP such as weight loss, gastrointestinal symptoms, and health related quality of life (HRQoL) in comparison to LRYGB among the patients with BMI 35–50. 

## 2. Materials and Methods

This prospective study was performed in the Surgery Department of Lithuanian University of Health Sciences, Kaunas, Lithuania, during the period between 2017 April and 2018 December. Patients were informed about both procedures and were allowed to choose the one they thought to be most appropriate. LGGCP was completely covered by the Lithuanian Health Insurance Fund, while patients who chose LRYGB had to pay for single-use instruments. All consecutive adult patients (age ≥ 18 y) who attended the Outpatient Clinic of the Surgery Department during the study period and had obesity (BMI between 35 and 50) were asked to participate in the study. Exclusion criteria were previous upper abdominal surgery and patients with end-stage organ failure, esophagitis grade C or D according to the Los Angeles classification, and Barrett’s oesophagus. Only patients who signed an informed consent were included in the study. All patients before surgery underwent clinical assessment, laboratory testing, and gastroscopy. 

**Figure 1:**
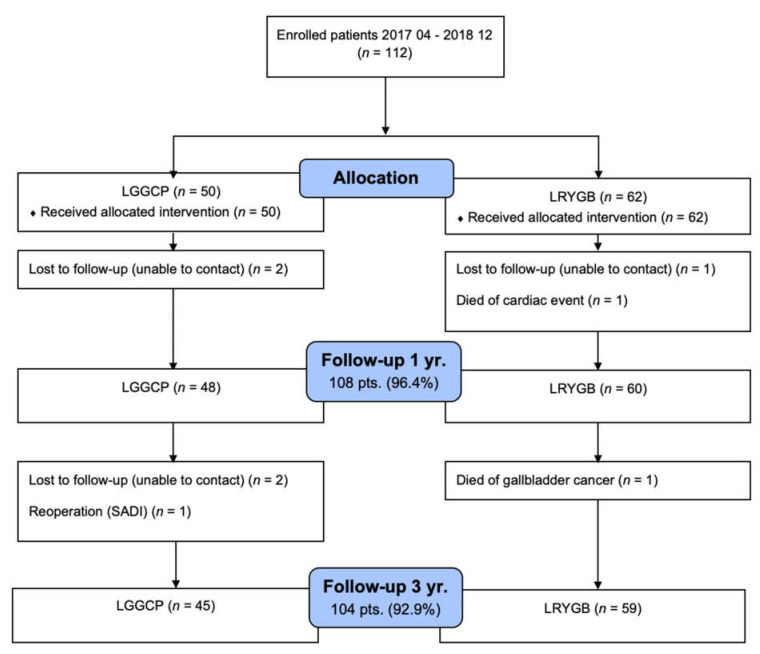
Postoperative patient follow-up.

Fifty patients had LGGCP, and sixty-two patients underwent LRYGB. Two patients died during the follow-up period leaving, 110 patients for further analysis. One patient died 9 mo after surgery from myocardial infarction; the other patient died from gallbladder cancer 2 year after the operation. One patient 2 year after LGGCP was converted to laparoscopic single anastomosis duodenal-ileal bypass (SADI) and was excluded from the study. Three patients were pregnant after LRYGB at 3 year follow-up, and their weight data, gastrointestinal symptoms score, and HRQoL were not included in the analysis. The follow-up rate was 96.4% and 92.9%, 1 year and 3 year after surgery, respectively (Figure 1).

### 2.1. Surgical Technique

All operations were performed laparoscopically and by the same surgical team. In LGGCP, gastric greater curvature was mobilised from the omentum starting 3–5 cm from the pylorus and all the way to the angle of His. The fundus was completely mobilised, and the left crus was exposed. In all cases, the hiatal region was explored for possible hernia. If a hiatal hernia was present, the right crus was approached through the hepatogastric ligament, and cruroraphy was performed with separate 2/0 Ethibond (Ethicon, Endo-Surgery) sutures. The 36 Fr tube was placed with the tip in the duodenum, and the imbrication of greater curvature was started from the fundal region with separate 2/0 Prolene (Ethicon, Endo-Surgery) sutures and finished 3–5 cm from the pylorus. The second row of sutures was placed in between the first row of sutures, further imbricating the greater curvature. This technique resulted in two-row plication. When the plication was complete, the gastric tube was retracted into the oesophagus and pushed down back into the stomach to test if there was severe narrowing of the lumen. In the case when the tube could not pass down into the prepyloric region, some stitches were released and replication performed. 

In LRYGB, a small proximal gastric pouch up to 30 mL was constructed with 45 mm linear staplers. An antecolic, antegastric gastrojejunostomy was performed approximately 3 cm in length with a linear stapler. The length of the biliopancreatic limb varied from 100 cm to 150 cm with a longer limb among the patients with type 2 diabetes mellitus (T2DM). A 120 cm alimentary limb was measured, and side-to-side anastomosis was performed between the afferent and efferent limbs with a linear stapler. The LRYGB construction was finished by dividing the afferent loop in between entero-enterostomy and gastroenterostomy. The leak test with methylene blue solution was routinely performed to check for leaks in the gastroenterostomy. Mesenteric defects were closed by continuous 2/0 Ethibond (Ethicon, Endo-Surgery) sutures in all patients.

The duration of the operation, complications, and the length of hospital stay were recorded during the postoperative period. The data from follow-up visits at 12 mo and 36 mo were included in the analysis. Preoperatively, gastroesophageal reflux disease (GERD) was diagnosed if patients had esophagitis on endoscopy or had used antacid medications for more than 2 mo [8]. The same criteria were also used to define GERD postoperatively. The gastroscopy after surgery was performed only if patients had newly onset GERD symptoms. Hypertension was diagnosed if blood pressure was ≥140/90 mmHg or the patients were on hypertensive medications. Remission of hypertension was considered if blood pressure was <140/90 mmHg without antihypertensive medications, and improvement was defined as a reduction in the number or dosage of antihypertensive medications [9]. T2DM was present if fasting plasma glucose levels were ≥7.0 mmoL/L or HbA1c ≥ 6.5% or patients were treated with peroral medications or insulin [10]. T2DM complete remission was present if HbA1c <6.0% and FPG <5.6 mmol/l without pharmacological therapy, and improvement was defined if the number or dosage of antidiabetic medications decreased [9]. Weight loss was presented as loss of absolute kilograms, percentage of excess BMI loss (%EBMIL), and %TWL. 

The self-administered 15-item Gastrointestinal Symptom Rating Scale (GSRS) questionnaire was used to evaluate the severity of gastrointestinal symptoms [11]. Each item was graded on a 7-point Likert scale, where 1 was no symptoms and 7 was the most severe symptoms. The mean values for diarrhoea, indigestion, constipation, abdominal pain, and reflux were estimated. The EQ-5D-3L questionnaire was used to obtain data about HRQoL (EQ-5D; http://www.euroqol.org, accessed on 24 March 2020). This questionnaire measures HRQoL in 5 dimensions: mobility, self-care, usual activities, pain /discomfort, and anxiety/depression. Every dimension is evaluated by the patient on a scale from 1 to 3, where 1 is the best possible outcome and 3 is the worst. At the end, a sequence of 5 numbers is produced for each patient. This sequence is then converted into health state utilities, which are scored between 0 and 1, where “1” is perfect health and “0” is death. Conversion data from the European population obtained by the VAS method were used as there were no EQ-5D-3L questionnaire validated data for the Lithuanian population [12]. Estimated health state utilities were used as a metric to quantify HRQoL after LGGCP and LRYGB.

### 2.2. Statistical Analysis

The analysis of the data was performed using the statistical software SPSS 27.0. The assumption of the normality of the quantitative variable was verified using the Kolmogorov–Smirnov test. Student’s (*t*) test was used to compare the normally distributed quantitative values of the two independent groups. The data are presented as the mean and standard deviation (SD). Abnormally distributed continuous and ordinal variables were compared with the Mann–Whitney *U*-test and are presented as the median with the interquartile range (25th–75th percentile). The Chi-squared test was used to compared categorical variables. The Pearson correlation coefficient was applied to evaluate the strength of the relationship between two quantitative traits, satisfying the conditions of the normal distribution. The differences and dependencies between values were considered statistically significant when the *p*-value was <0.05.

## 3. Results

There were no significant differences between the groups regarding baseline characteristics such as sex, age, BMI, and comorbidities (Table 1). The average operation time was similar in the LGGCP and LRYGB groups, 78.20 (16.56) and 74.92 (15.85) min, respectively. Pearson correlation analysis showed no significant relationship between patients’ BMI and the duration of surgery (LGGCP—r = 0.047, *p* = 0.745; LRYGB—r = 0.174, *p* = 0.180). The average hospital stay was 2.36 (0.95) d in the LGGCP group and 2.16 (0.41) d in the LRYGB group (*p* = 0.800). Postoperative complications were observed in two patients (4%) in the LGGCP group and one patient (1.6%) in the LRYGB group. Both patients in the LGGCP group experienced early complications—nausea and vomiting. A patient in the LRYGB group had bleeding from the gastroenterostomy (treated conservatively). Two (3.2%) patients in the LRYGB group developed anastomotic ulcers within 1 year after surgery. One (1.6%) of them underwent surgery due to ulcer perforation. No late complications were observed in the LGGCP group. There was no statistically significant difference in the early and late complications between the groups (*p* = 0.811 and *p* = 0.331, respectively).

**Figure 2:**
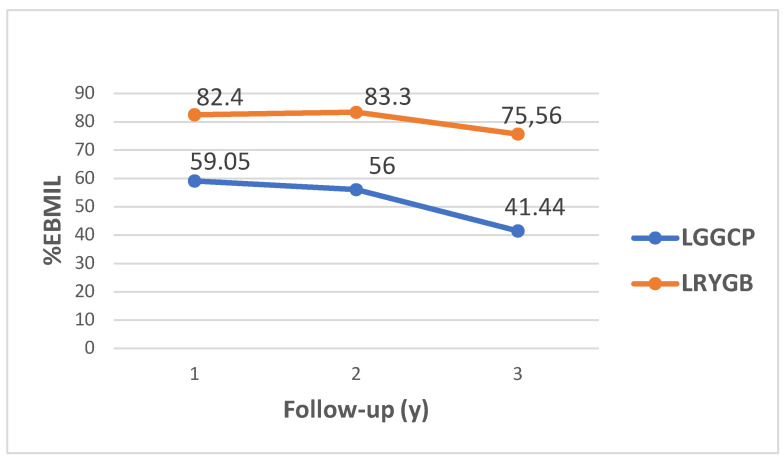
Mean %EBMIL after LGGCP and LRYGB at 1 year, 2 year and 3 year.

The mean %EBMIL 1 year after surgery was 59.05 (25.34) in the LGGCP group and 82.40 (19.03) in the LRYGB group (*p* < 0.001) and 3 year after was 41.44 (26.74) and 75.59 (19.14), respectively (*p* < 0.001) (Figure 2). Patients who underwent LRYGB achieved significantly greater weight and BMI loss compared to the patients who underwent LGGCP (Table 2). Thirty-six (78.3%) patients in the LGGCP group and twenty-five (41.7%) in the LRYGB group regained more than 15% from their nadir weight, 3 year after surgery. The median %WR after LGGCP was 31.53 (25th–75th percentile, 15.69–57.33) and after LRYGB was 12.16 (25th–75th percentile, 5.4–21.72) (*p* < 0.001).

Thirty-six patients in the LGGCP group and thirty-nine in the LRYGB group had hypertension, and the data about thirty-two (88.9%) and thirty-six (94.7%) patients, respectively, were available 3 year after surgery. Nineteen (59.4%) in the LGGCP group and twenty-five (69.4%) in the LRYGB group achieved remission (Table 3). Preoperatively, six patients in the LGGCP group and twelve patients in the LRYGB group had T2DM, and the data about five (83.3%) and eleven (91.7%) patients, respectively, were available 3 year after surgery. Complete remission was observed in three (60%) patients in the LGGCP group, which were treated with oral antidiabetic medications (OAD) before surgery, and nine (81.8%) patients in the LRYGB group (Table 4). Both techniques had a statistically significant effect on the remission of hypertension and T2DM, but there was no significant difference in the remission of comorbidities between the groups.

Preoperatively, GERD was present in 63 patients, 30 in the LGGCP group and 33 in the LRYGB group. Twelve (40%) continued to have GERD, and six (30%) developed GERD de novo up to 3 year after LGGCP, while after LRYGB, the respective numbers of patients were three (9%) and two (7.4%). Patients who underwent LRYGB had a significantly lower prevalence of GERD compared to patients who underwent LGGCP (Table 5).

The scores of all gastrointestinal symptoms measured by the GSRS questionnaire significantly decreased 3 year after both procedures, except reflux after LGGCP (Table 6). Patients 3 year after LRYGB had a significantly higher abdominal pain score as compared to patients after LGGCP, and LGGCP resulted in significantly more GERD symptoms.

After surgery, the quality of life of both groups improved statistically significantly in such dimensions as mobility, self-care, usual activities, and pain/discomfort. There was no statistically significant change in anxiety/depression. There was no difference in HRQoL between groups after 1 year (*p* = 0.247), but 3 year after surgery, it was significantly better in the LRYGB group (*p* < 0.001) (Table 7). Patients with %WR > 15 had significantly lower HRQoL 3 year after surgery, 0.783 (25th–75th percentile, 0.690–1.0) vs. 1.0 (25th–75th percentile, 0.783–1.0), *p* = 0.003. 

## 4. Discussion

This prospective study compared two surgical procedures, LGGCP and LRYGB, in the treatment of patients with obesity. LGGCP patients 3 year after surgery lost significantly less weight, had less abdominal pain, more reflux symptoms, and a lower quality of life. 

The groups were similar regarding age, sex, preoperative BMI, and distribution of comorbidities. The patients after LRYGB lost significantly more weight after 1 year, and the difference was even more evident at 3 year follow-up. %EBMIL 3 year after LGGCP was 41.4, similar to the results from our previous study [13]. There was a significantly higher percentage of weight regain in the LGGCP group 3 year after the procedure. It was shown by CT volumetry that the volume of the stomach after LGGCP enlarged and could reach on average 845 mL 3 year after surgery [14]. This could be due to the shrinkage of the imbricated part of the stomach with the following dilatation of the stomach tube, or in 40–50% of the cases, the sutures may cut through the stomach wall and result in unfolding of the inverted part of the stomach [13,14]. Stomach enlargement may result in more weight regain as the restrictive effect of the LGGCP operation is lost.

During the early postoperative period, the LGGCP group had more abdominal symptoms such as abdominal pain, nausea, or vomiting. These are a widely recognised complications after LGGCP and are responsible for the prolonged hospital stay. Sometimes, patients even need reoperation after LGGCP due to gastric obstruction [15]. In contrast, 3 year after surgery, LRYGB patients had higher score of abdominal pain measured by the GSRS questionnaire, even though abdominal pain significantly decreased after LRYGB as compared to the preoperative level. The indigestion, diarrhoea, and constipation scores were similar between the two procedures and improved significantly as compared to the baseline values. The result from the present study is in a contrast with the findings of Chahal-Kummen M et al. [16], where the increase in all GSRS scores except for reflux was observed 2 year after RYGB.

GERD symptoms preoperatively were present in more than half of the patients in both groups. Three years after LGGCP, forty percent of patients retained GERD symptoms and thirty percent had de novo GERD, while LRYGB significantly reduced the GERD symptoms. In the present study, 8.5% of patients after LRYGB experienced GERD symptoms, a number that is similar to the 8.1% prevalence of GERD after RYGB found in the recent German Bariatric Surgery Registry study [17]. LGGCP is known to produce more GERD symptoms, though a recent study showed that endoscopic signs of GERD were less prevalent after LGGCP as compared to SG, 4.8% and 19.2%, respectively. Higher prevalence of GERD after LGGCP in our population could be related to the fact that in the present study, the definition of GERD included both endoscopic findings of esophagitis and/or treatment with proton pump inhibitors (PPIs). 

There were no differences in the remission of comorbidities such as hypertension and T2DM between the groups in the current study. By contrast, Casajoana A et al. [7] in a randomised study comparing metabolic RYGB, SG, and GGCP found that the complete remission rate of T2DM 5 year after the operations was 46.7%, 20.0%, and 6.6% respectively. The complete remission rate after metabolic RYGB in Casajoana A et al.’s [7] study decreased from 80% after 1 year to 46.7% after 5 year. In our study, the complete remission of T2DM 3 year after LRYGB reached 81.8%. However, the data from the present study showed that LGGCP resulted in a higher 60% complete T2DM remission rate, and the only difference between LRYGB and LGGCP was among the patients who used insulin to control glycaemia. No one in the LGGCP group who had T2DM treated with insulin achieved complete remission, in line with the findings from our previous study [12]. One of the factors that may explain such a difference is postprandial GLP-1 secretion, which was found to be higher after LRYGB compared to LGGCP [7]. Remission or improvement of hypertension was observed in all patients after LRYGB and in 87.5% of cases after LGCCP. Similar partial/complete remission rates of hypertension were presented in a recent systematic review analysing LGCCP results [4]. 

HRQoL after both procedures improved significantly up to 3 year after surgery. However, in the LGCCP group, HRQoL decreased, while LRYGB patients retained approximately the same level of HRQoL. Three years after surgery, a significant difference between the procedures emerged in favour of LRYGB. One possible explanation could be the higher rate of weight regain after LGGCP, as weight regain was shown to be related to quality of life [18]. It must be noted that even lower weight loss after LGGCP had a significant impact on HRQoL. Despite weight regain, other factors such as moderate to severe (GSRS score ≥ 3) abdominal pain and indigestion may influence quality of life after RYGB [19]. In the present study, patients after LRYGB were able to maintain very good HRQoL 3 year post-surgery. This could be related to nonsignificant weight regain and low GSRS scores of abdominal pain and indigestion after LRYGB during the follow-up period in our study population. 

Revisional surgery is rather common after LGGCP and is reported to be within a limit of 5.5% to 32.4% [13,20] up to 5 year after intervention. In the present study, only one patient underwent conversion to SADI, much less than in other studies. The reason for this underrepresentation of revisional procedures could be the fact that we offered only replication or SADI as a completely free reoperation and that despite worse weight loss after LGGCP within 3 year, still, patients had a reasonably good control of their comorbidities and improved HRQoL. 

This study had some limitations. First, it was a prospective non-randomised study where the patients were allowed to choose the procedure. The patients who chose LRYGB had to pay additionally for single-use instruments. This could have had some selection bias because the patients were assumed to be wealthier in the LRYGB group, though we did not collect data on income. Some studies demonstrated that patients with a low income lose less weight after bariatric surgery [21,22]. However, the short- and mid-term weight loss difference between low- and mid–high-income groups in Chen JC et al.’s study [21] was no higher than 5 %EBMIL. The difference in %EBMIL between LRYGB and LGGCP after 3 year in our study was 34%, far beyond the possible impact of income on weight loss. Second, the 3 year follow-up was performed during the COVID-19 pandemic from 2020 to 2021. The lockdowns, working from home, and higher level of anxiety in society could have had an impact on the lower %EBMIL and weight regain 3 year after the surgical procedures [23]. Third, the study was underpowered to detect differences in the resolution of comorbidities, especially T2DM. Finally, HRQoL was measured by a questionnaire that was not disease specific and was based on indirect patient preferences. Such a questionnaire was chosen because we wanted to perform a cost–utility analysis. The National Institute for Health and Care Excellence (NICE) supports the use of the EQ-5D in adults to measure HRQoL [24]. However, there are no validated EQ-5D-3L scores for the Lithuanian population. We used scores that were estimated from the more general European population. Recent meta-analysis found a similar increase in EQ-5D-3L scores 1 year after bariatric surgery, as was found in our study, supporting the relevance of our choice to estimate the EQ-5D-3L scores [25]. 

## 5. Conclusions

Three years after surgery, LGGCP patients lost significantly less weight, had less abdominal pain, more reflux symptoms, and a lower quality of life compared to LRYGB patients.

## Figures and Tables

**Table 1:** Baseline characteristics of the patients included in the study.

	LGGCP *n* = 50	LRYGB *n* = 62	*p*-Value
**Sex F/M**	41/9	47/15	0.492
**Age year, mean (SD)**	46.28 (11.53)	44.76 (11.60)	0.490
**BMI, mean (SD)**	42.60 (4.17)	42.86 (3.78)	0.738
**Weight kg, mean (SD)**	121.44 (16.88)	126.50 (18.09)	0.132
**Hypertension, *n* (%)**	36 (72.0)	39 (62.9)	0.309
**Type 2 diabetes mellitus, *n* (%)**	6 (12.0)	12 (19.35)	0.292

**Table 2:** Comparison of the weight and BMI 1 year, 2 year and 3 year after LRYGB and LGGCP.

Outcome	LGGCP (*n* = 48 at 1 Year, *n* = 45 at 3 Year)	LRYGB (*n* = 60 at 1 Year, *n* = 56 at 3 Year)	*p*-Value Baseline vs.		*p*-Value LGGCP vs. LRYGB
			LGGCP	LRYGB	
**Mean weight, kg (SD)**					
**Baseline**	-	-			0.132
**1 year**	92.08 (16.12)	83.47 (13.74)	<0.001	<0.001	0.003
**2 year**	93.39 (15.65)	82.87 (13.93)	<0.001	<0.001	<0.001
**3 year**	101 (18.82)	87.31 (15.15)	<0.001	<0.001	<0.001
**Mean BMI, kg/m^2^ (SD)**					
**Baseline**	42.60 (4.17)	42.86 (3.78)			0.738
**1 year**	32.43 (5.04)	28.36 (3.60)	<0.001	<0.001	<0.001
**2 year**	32.93 (4.92)	28.12 (3.42)	<0.001	<0.001	<0.001
**3 year**	35.47 (5.71)	29.70 (3.83)	<0.001	<0.001	<0.001
**%TWL (SD)**					
**1 year**	23.61 (9.73)	33.58 (7.62)			<0.001
**2 year**	19.51 (9.23)	34.05 (8.05)			<0.001
**3 year**	16.51 (10.64)	30.43 (8.80)			<0.001
**%EBMIL (SD)**					
**1 year**	59.05 (25.34)	82.40 (19.03)			<0.001
**2 year**	56.00 (24.34)	83.30 (19.20)			<0.001
**3 year**	41.44 (26.74)	75.56 (19.14)			<0.001

**Table 3:** Hypertension remission and improvement 3 year after operation.

	LGGCP *n* = 32	LRYGB *n* = 36	*p*-Value
	*n* (%)	*n* (%)	
Remission *	19 (59.4)	25 (69.4)	0.247
Improvement **	9 (28.1)	11 (30.6)	
No change	3 (9.4)	-	
Worse	1 (3.1)	-	

* Remission—normal blood pressure (<140/90 mmHg) without medications. ** Improvement—number or dosage of antihypertensive medication decreased, or blood pressure levels decreased with the same antihypertensive treatment.

**Table 4:** Diabetes mellitus remission and improvement 3 year after the operation according to the T2DM treatment before surgery.

	LGGCP *n* = 5		LRYGB *n* = 11		*p*-Value
T2DM Treatment before Surgery	OAD Medications, *n* (%)	Insulin or Insulin with OAD Medications, *n* (%)	OAD Medications, *n* (%)	Insulin or Insulin with OAD Medications, *n* (%)	
Complete remission *	3 (60)	-	7 (63.6)	2 (18.2)	1.000
Improvement	-	1 (20)	-	2 (18.2)	
No change	1 (20)	-	-	-	

OAD—oral antidiabetic medications. * Complete remission—HbA1c < 6.0% and FPG < 5.6 mmoL/L without pharmacological therapy.

**Table 5:** GERD before and after surgery.

	Before Surgery		1 Year after Surgery		3 Year after Surgery	
	Typical GERD Symptoms	No GERD Symptoms	Persistence of GERD Symptoms	New Onset of GERD Symptoms	Persistence of GERD Symptoms	New Onset of GERD Symptoms
LGGCP	30 (60%)	20 (40%)	15 (50%)	4 (20%)	12 (40%)	6 (30%)
LRYGB	33 (55%)	27 (45%)	5 (15%)	1 (3.7%)	3 (9%)	2 (7.4%)
*p*-value	0.566		<0.001		<0.001	

**Table 6:** Distribution of results of the Gastrointestinal Symptom Rating Scale before and 1 year and 3 year after surgery.

	LGGCP *n* = 45	LRYGB *n* = 56	*p*-Value Baseline vs.		*p*-Value *
Abdominal Pain	Mean; Median (25th–75th Percentile)	Mean; Median (25th–75th Percentile)	LGGCP	LRYGB	
Before	1.79; 1.67 (1.0–2.0)	1.80; 1.67 (1.0–2.0)			0.981
1 year after	1.44; 1.0 (1.0–1.66)	1.36; 1.0 (1.0–1.83)	0.003	<0.001	0.824
3 year after	1.01; 1.0 (1.0–1.0)	1.20; 1.0 (1.0–1.33)	<0.001	<0.001	<0.001
**Reflux**					
Before	2.03; 1.5 (1.0–2.5)	1.81; 1.5 (1.0–2.5)			0.557
1 year after	1.82; 1.0 (1.0–2.5)	1.13; 1.0 (1.0–1.0)	0.246	<0.001	<0.001
3 year after	1.79; 1.25 (1.0–2.5)	1.18; 1.0 (1.0–1.0)	0.432	<0.001	<0.001
**Indigestion**					
Before	2.34; 2.25 (1.75–3.0)	2.34; 2.25 (1.75–3.0)			0.104
1 year after	1.68; 1.75 (1.0–2.0)	1.68; 1.75 (1.0–2.0)	<0.001	0.950	0.098
3 year after	1.39; 1.5 (1.0–1.75)	1.39; 1.5 (1.0–1.75)	<0.001	<0.001	0.461
**Diarrhoea**					
Before	1.67; 1.0 (1.0–1.92)	1.67; 1.33 (1.0–2.0)			0.555
1 year after	1.40; 1.0 (1.0–1.66)	1.62; 1.33 (1.0–2.0)	0.286	0.914	0.076
3 year after	1.16; 1.0 (1.0–1.0)	1.20; 1.0 (1.0–1.0)	0.015	0.001	0.479
**Constipation**					
Before	1.89; 1.33 (1.0–2.33)	1.97; 1.67 (1.0–2.33)			0.601
1 year after	1.68; 1.33 (1.0–2.0)	1.37; 1.0 (1.0–1.66)	0.367	<0.001	0.057
3 year after	1.26; 1.0 (1.0–1.33)	1.21; 1.0 (1.0–1.33)	0.005	<0.001	0.313

* *p*-value of the nonparametric test comparing the results of the groups with each other.

**Table 7:** Mean EQ-5D-3L index values before and after surgeries.

Outcome	EQ-5D-3L Index, Mean; Median (25th–75th Percentile)		*p*-value Baseline vs.		*p*-Value
	**LGGCP**	**LRYGB**	**LGGCP**	**LRYGB**	
**Before**	0.636; 0.639 (0.500–0.761)	0.707; 0.715 (0.496–1.000)			0.091
**After 1 year**	0.859; 1.000 (0.757–1.000)	0.898; 1.000 (0.783–1.000)	<0.001	<0.001	0.247
**After 3 year**	0.762; 0.779 (0.690–0.794)	0.898; 1.000 (0.783–1.000)	0.003	<0.001	<0.001
